# Education Research: Evaluation of Epilepsy Surgery Education in Epilepsy and Clinical Neurophysiology Fellowship Programs

**DOI:** 10.1212/NE9.0000000000200018

**Published:** 2022-11-15

**Authors:** Hernan Nicolas Lemus, Barbara A. Dworetzky, Ellen J. Bubrick, Garth Rees Cosgrove, Steven Tobochnik

**Affiliations:** From the Departments of Neurology (H.N.L., B.A.D., E.J.B., S.T.) and Neurosurgery (G.R.C.), Brigham and Women's Hospital, Boston, MA.

## Abstract

**Background and Objectives:**

To assess the scope of surgical epilepsy exposure and training among fellows in Epilepsy and Clinical Neurophysiology (CNP) fellowship programs in the United States. Characteristics associated with increased fellow involvement in epilepsy surgery were evaluated.

**Methods:**

A 10-question multiple-choice survey was designed to characterize individual fellowship programs, epilepsy surgery programs, trainee involvement, and assessment of trainee performance. The survey was distributed to program directors of adult Epilepsy and CNP-EEG track fellowships between November 2021 and April 2022. Epilepsy surgery procedures included resective approaches, neurostimulation modalities, and palliative interventions approved for drug-resistant epilepsy. Associations between categorical variables were evaluated using the Fisher exact test.

**Results:**

There were 37 responses from a total of 72 survey recipients (51% response rate). The majority (68%) of surgical programs performed >30 surgical procedures per year. The range of procedures was overall similar across programs. At most programs, fellows were personally involved in 1–10 (49%) or 11–30 (46%) surgical procedures per year. Institutions with >50 surgical cases/year were more likely to expose fellows to >10 cases/y compared with institutions with volumes ≤50 per year (77% vs 33%, *p* = 0.017). Fellows had the greatest involvement in presurgical planning with more variable involvement in perioperative and postoperative activities. Competency in surgical management was primarily investigated through faculty assessments (97%), whereas oral (46%) and written (30%) assessments were less frequently used.

**Discussion:**

High-volume epilepsy surgery centers provide trainees with increased exposure despite also having more fellowship positions. There is variability in surgical epilepsy exposure, trainee involvement, and performance evaluation metrics between institutions. We identify specific areas that programs may focus on to improve fellow competency in the surgical management of epilepsy.

Seizures are refractory to medications in about 30% of the patients with epilepsy, which represents over a million people living in the United States with drug-resistant epilepsy (DRE).^1,2^ Epilepsy surgery is an effective option for many people with DRE^3,4^; however, it remains significantly underutilized. Despite an increase in the number of specialized epilepsy centers between 2003 and 2012, the number of resective surgeries has decreased.^5^ Recognition of patients with epilepsy syndromes amenable to surgical intervention and early referral to specialized epilepsy centers is necessary to improve outcomes in DRE.^6,7^ Furthermore, recent advances in epilepsy surgery, including laser ablation therapies or implantation of neurostimulation devices, have now become potential options for patients who are not candidates for or wish to avoid resective surgery. Because specialty-trained neurologists serve an integral role in facilitating the identification and presurgical workup of epilepsy surgery candidates, it is necessary to critically evaluate the exposure and assessment methodology in surgical epilepsy management received by trainees during their fellowship.

The Accreditation Council for Graduate Medical Education (ACGME) Epilepsy milestones indicate that an epilepsy fellow must (1) plan and manage phase 1 (scalp video-EEG monitoring) and phase 2 (invasive EEG monitoring) surgical evaluation, (2) synthesize presurgical data to establish a surgical management plan, (3) manage neurologic issues in the postoperative patient, and (4) identify candidates for use of medical devices approved for the treatment of DRE.^8^ A trainee is required to be the primary reviewer of at least 5 intracranial studies including the use of subdural electrodes, depth electrodes, and intraoperative electrocorticography.^9^ Similarly, the ACGME clinical neurophysiology (CNP) milestones indicate that a CNP fellow must demonstrate detailed knowledge of advanced treatment options for medically refractory epilepsy.^10^ Prior studies have shown deficiencies and lack of consistency in the education of neurology residents to achieve the required milestones for EEG interpretation.^11,12^ Notably, there is scarce literature assessing the quality of surgical education in epilepsy and CNP trainees.

The current study investigated the scope of surgical epilepsy exposure and training among fellows in Epilepsy and CNP fellowship programs in the United States. We hypothesized that there is a lack of standardized surgical epilepsy training and assessment among the different ACGME-accredited fellowship programs. By examining these trends, we aim to motivate educational initiatives to better prepare trainees to identify and treat surgically remediable epilepsy syndromes and by extension improve access to surgical treatment for people with DRE.

## Methods

### Study Design and Population

This study involved a national survey of institutions with epilepsy and/or CNP fellowship programs. A search of CNP-EEG track and Epilepsy ACGME-accredited programs was conducted by state. Pediatric epilepsy programs and CNP programs with a preference for an EMG track, autonomic track, or neurophysiologic intraoperative monitoring were excluded. The American Academy of Neurology member directory was queried to obtain the email of the program director. Between November 2021 and April 2022, a request to complete the survey was sent by email to eligible program directors with instructions and a link to the survey. A 1-time reminder was sent after 4 weeks if no response was received. Each program director was quantified as a single respondent. Two institutions had responses included from 2 program directors who separately evaluated the Epilepsy and CNP programs.

### Survey Design

A multiple-choice question survey was designed to broadly evaluate the characteristics of each fellowship program institution, its epilepsy surgery program, trainee exposure, and performance assessments. A pilot survey was initially sent to one epilepsy program director, one CNP program director, and one neurosurgeon to review validity of questions and responses; their feedback was used for final survey development. The institutional characteristics included the geographic region, the National Association of Epilepsy Centers (NAEC) classification level, and the number of ACGME-accredited fellows per year of training. The surgical program characteristics included the type and volume of surgical procedures performed, including common resective approaches, neuromodulation, and palliative interventions for DRE^13^ (eData, links.lww.com/NE9/A4). Trainee exposure was investigated by the level of involvement in surgical workflow and volume of cases exposed to. Trainee education and assessment were investigated by the types of didactic resources and methods of measuring fellows' competency in epilepsy surgical management. The survey contained a total of 10 questions (eData, links.lww.com/NE9/A4), designed to take less than 5 minutes to complete to maximize participation. A publicly available survey platform was used to design, record, and aggregate responses (Google Forms: docs.google.com/forms/).

### Statistical Analysis

Data preprocessing and descriptive statistics were performed using R 4.0.3 (R Foundation for Statistical Computing, Vienna, Austria, R-project.org/). Associations between categorical variables were evaluated using the Fisher exact test. All statistical tests were 2 tailed with a significance level of 0.05.

### Standard Protocol Approvals, Registrations, and Patient Consents

This study was exempt from Institutional Review Board review and did not meet the criteria for human subject research.

### Data Availability

The principal author has full access to all the data; the author has the right to publish all the data separate and apart from any sponsor.

## Results

### Institutional Characteristics

**Figure 1:**
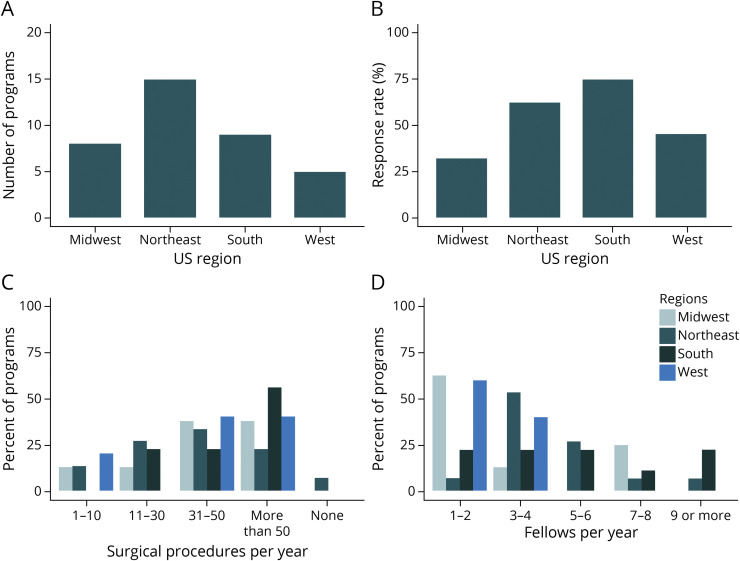
Demographics and Characteristics of Epilepsy Centers Surveyed (A) Total number of programs surveyed per US region. (B) Response rate of programs surveyed per region. (C) Number of surgical procedures performed per year per region; the Y axis corresponds to the percent of the responding programs. (D) Number of fellows per program by region; the Y axis corresponds to the percent of the responding programs.

Program directors from 37/72 (51%) institutions with adult epilepsy and/or CNP-EEG fellowship programs responded to the survey. Fellowship program demographics are shown in Table. There was a fair representation of the programs across the United States, ranging from 5 (14%) programs in the Western region to 15 (41%) in the Northeast region, with a geographic response rate ranging from 32% in the Midwest region to 75% in the Southern region (Table, Figure 1, A–D). The majority (92%) of responses were received from NAEC level 4 centers.^5^

**Table:** Demographic Characteristics of Surveyed Programs (N = 37)

Characteristic	N (%)
Fellowship type	
Epilepsy	12 (32)
CNP	3 (8)
Both	22 (60)
NAEC classification	
Level 3	1 (3)
Level 4	34 (92)
I do not work in a level 3 or level 4	2 (5)
US region	
Northeast	15 (40)
West	5 (14)
Midwest	8 (22)
South	9 (24)
No. of ACGME fellows trained per year	
1–2	11 (30)
3–4	13 (35)
5–6	6 (16)
7–8	4 (11)
9 or more	3 (8)

Abbreviations: ACGME = Accreditation Council for Graduate Medical Education; CNP = clinical neurophysiology; NAEC = National Association of Epilepsy Centers.

### Surgical Program Characteristics

**Figure 2:**
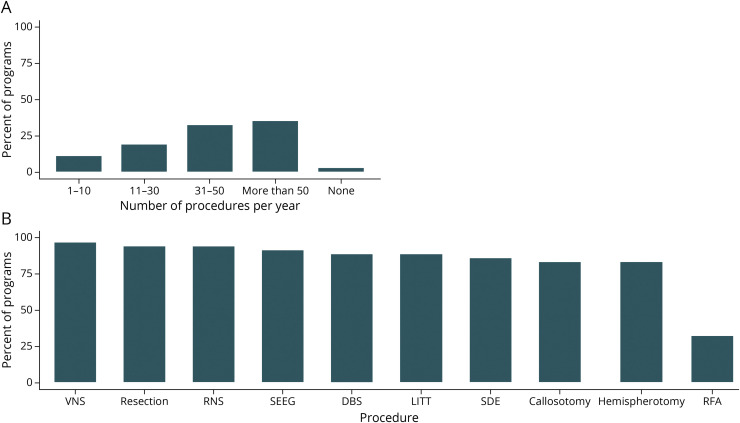
Characteristics of Surgical Procedures Performed at Responding Centers (A) Total number of surgical procedures performed per year per institution. (B) Types of procedures performed. DBS = deep brain stimulation; LITT = laser interstitial thermal therapy; RNS = responsive neurostimulation; SDE = subdural electrode implantation = RFA, radiofrequency ablation; SEEG = stereoelectroencephalography; VNS = vagus nerve stimulation.

The majority (68%) of surgical programs performed >30 surgical procedures per year (Figure 2A). High-volume centers were surveyed across each geographic region, with >50 procedures per year reported by 3/8 (38%) Midwest, 3/15 (20%) Northeast, 5/9 (56%) South, and 2/5 (40%) West region programs. Institutions performing >50 procedures per year had more ACGME-accredited fellows than institutions performing ≤50/y (69% vs 17% with ≥5 fellows, respectively, *p* = 0.003).

The range of surgical procedures was overall similar across programs (Figure 2B). Radiofrequency ablation was least frequently performed (32%). Geographically, there was a trend toward fewer subdural electrode implantation procedures offered in the Midwest compared with the rest of the country (63% Midwest institutions vs 93% other regions, *p* = 0.056).

### Trainee Exposure to Epilepsy Surgery Procedures

**Figure 3:**
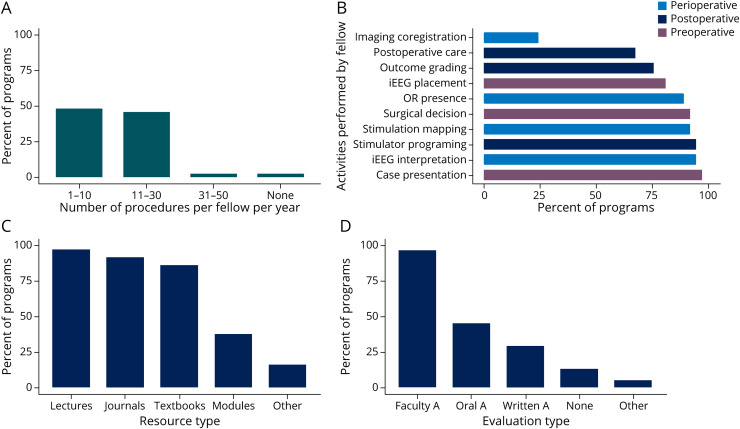
Trainee Exposure and Assessments in Epilepsy Surgery (A) Number of procedures per year in which trainees are personally involved. (B) Types of preoperative, perioperative, and postoperative activities that trainees are involved in. (C) Types of didactic resources used by surveyed programs. (D) Types of assessments used to assess competency in epilepsy surgery management. Imaging coregistration = performing multimodal imaging coregistration for intracranial EEG. iEEG = intracranial EEG recording; stimulation mapping = performing electrical stimulation mapping; iEEG placement = determination of electrode/lead placement; Faculty A = faculty assessments; Oral A = oral assessments; Written A = Written Assessments.

At most programs, each fellow was involved in 1–10 (49%) or 11 to 30 (46%) surgical procedures per year (Figure 3A). Institutions with >50 surgical cases per year were more likely to expose fellows to >10 cases per year compared with institutions with volumes ≤50 per year (77% vs 33%, *p* = 0.017). Exposure to >10 cases per year was similar between programs with <5 and ≥5 fellows (42% vs 62%, *p* = 0.313). Compared with programs with combined CNP and epilepsy accreditations, there was a trend toward greater volume of exposure per fellow in programs with only epilepsy accreditation (>10 cases/y in 75% vs 36%, *p* = 0.071). Rates of exposure were similar across regions, with >10 cases per year reported by 5/8 (63%) Midwest, 5/15 (33%) Northeast, 4/9 (44%) South, and 4/5 (80%) West region programs.

The rates of trainee involvement in preoperative, perioperative, and postoperative activities are shown in Figure 3B. Fellow involvement in preoperative activities ranged from 97% participation in case presentations to 81% participation in intracranial EEG planning. There were high rates of perioperative involvement in OR attendance (89%), intracranial EEG interpretation (95%), and stimulation/mapping procedures (92%), however, with a low rate of participation in multimodal electrode localization (24%). Postoperatively, fellows frequently participated in neurostimulator programming (95%) and less often in surgical outcome evaluation (76%) and postoperative care (68%).

### Didactic Resources and Evaluation

Didactic resources used for surgical epilepsy education predominately included faculty lectures, textbooks, and journal articles (Figure 3C). Online resources were used by a minority (38%) of the programs. Competency in surgical management was most often determined through faculty assessments (97%), whereas oral (46%) and written (30%) assessments were less frequently used (Figure 3D). A minority (14%) of the programs reported no formal assessment method.

## Discussion

This study sought to assess the scope of surgical epilepsy exposure and training provided by epilepsy and CNP-EEG programs in the United States by querying fellowship program directors. The results reveal variability in surgical exposure, fellow involvement, and performance assessments among programs. These data indicate that high-volume epilepsy surgery centers performing >50 cases per year provide trainees with an increased case volume of exposure, despite also having more ACGME-accredited fellowship positions.

The survey responses were heavily biased toward NAEC level 4 epilepsy centers, those that provide the highest level of diagnostic and surgical treatment options for people with DRE.^14^ Therefore, as expected, the range of procedures offered at these institutions was similar without significant geographic variation. However, despite similar exposure to different types of surgical procedures, this study identified specific areas with variability in fellow involvement, which programs may target to improve the knowledge base and competency of trainees. These included participation in postimplant multimodal electrode localization, postoperative management, and evaluation of surgical outcomes. Given the increasing use of stereotactic depth electrodes and high level of involvement of trainees in intracranial EEG interpretation and stimulation mapping procedures, an accurate understanding of electrode localization is critical. Similarly, the ACGME Epilepsy milestone focused on managing postoperative neurologic issues requires detailed knowledge of the structure and function of implanted and resected brain regions.

The training and education goals of fellowship should be achievable within the duration of the program, typically 1–2 years. With regard to the surgical management of epilepsy, fellows require a sufficient breadth of exposure to presurgical workup, perioperative management, and postsurgical follow-up. It is often challenging, however, for fellows to follow cases from presurgical evaluation through to the determination of long-term outcomes. Alternative strategies may be useful to address this issue, such as standardized postoperative review of cases to evaluate surgical complications and seizure outcomes. Similarly, online teaching modules were infrequently used, yet have proven to be effective resources for residency trainees to improve EEG interpretation abilities.^15^ There is a need for high-quality and versatile standardized training methods (e.g., online modules) for intracranial EEG, which could serve as an educational resource at sites with a lower volume of surgical cases or at times when elective surgeries decrease.^16^

Overall, there was a lack of consistency in the didactic resources and assessment methods to determine competency in surgical epilepsy management. A similar issue has been observed with regard to neurophysiology competency during neurology residency training; despite similar education methods across programs, about a third of senior residents lack confidence in EEG/EMG interpretation, and up to a quarter are felt by program directors not meet ACGME milestones.^11^ A lack of standardized training in EEG may contribute to misinterpretation of normal variants and misdiagnosis of epilepsy.^17^ This is a critical issue because up to 30% of patients who receive a diagnosis of epilepsy and do not respond to initial antiseizure medications may not have epilepsy.^18^ Similarly, the lack of standardized approaches to surgical epilepsy education raises concern for similar deficiencies in the diagnosis and management of epilepsy surgical care.

Almost all programs rely on faculty assessments to determine the competency of trainees, although, at times, these are supplemented with written and/or oral assessments. Faculty assessments have a myriad of biases and may have poor validity due to rating only select factors.^19^ In addition to increasing objective performance metrics, there is a need to diversify the methods used to evaluate fellows' skills in surgical epilepsy. The American Epilepsy Society offers online modules and self-assessments that may be used by program directors to enhance the competency of trainees in surgical epilepsy.^20,21^ Currently, the program directors of ACGME-accredited programs in the United States may also use the American Board of Psychiatry and Neurology (ABPN) Certification in epilepsy medicine, a standardized test that may identify areas of improvement.^22^ However, the surgical epilepsy content of the questions is focused on a limited number of treatments. An alternative assessment, the Epilepsy Fellowship In-service Training Examination, is a standardized examination used to target areas of improvement among epilepsy program directors; similarly, the examination content is based on the ABPN content examination.^23^ There is a need for national societies to create additional standardized examinations that may be used at a national level and include novel treatments such as neuromodulation or assess the interpretation of intracranial EEG. A revision to the ACGME-accredited epilepsy milestones was recently completed to improve the assessment of fellows.^24^ In this framework, surgical competency was divided into 3 areas, one focused on surgical planning, a second focusing on neuromodulation, and a third on communication to relatives and patients. The updated goals should serve as a guide for program directors when assessing the competency of the Epilepsy and CNP fellows. Similarly, there is a need to update the current CNP guidelines^10^ and specify the competencies a fellow must achieve in regards to the surgical management of patients with epilepsy.

This study has limitations. The survey response rate of 51% was adequate to achieve representation of training programs nationally; however, it remained biased toward NAEC level 4 epilepsy centers in the Northeast region and possibly toward larger-volume surgical centers. Nevertheless, this response rate is comparable to prior surveys of epilepsy surgery programs.^25,26^ The study did not assess pediatric programs or those programs that were not ACGME accredited. The cross-sectional nature of the study and the timing of the survey during a pandemic could have influenced the responses and number of surgical procedures performed at each program, although participants were instructed to answer based on their practices in a typical year. The survey tool was designed to maximize participation, and therefore, only categorical multiple-choice questions were included, which limited the granularity of the results and necessarily introduced somewhat arbitrary boundaries between categories. Despite this limitation and lack of previously validated query items in this research area, the answer choices allowed for adequate separation of training programs to identify meaningful trends and associations. For example, the groupings used in this survey to capture surgical volume and the separation in responses align with institutional volumes reported previously.^27^ Only standardized objective responses from program directors were elicited, and therefore, unique aspects of each training program (for instance, 1- vs 2-year programs) and trainee perceptions were not considered in this analysis. Additional future studies considering institution-specific curricula, temporal trends in surgery/trainee volume, trainee's outcomes (perception of surgical exposure, board certification, and level of surgical involvement posttraining), and validation of objective performance metrics with a control group are needed.

In conclusion, this national survey of Epilepsy and CNP fellowship programs revealed substantial variability in surgical epilepsy exposure, trainee involvement, and performance assessment metrics between institutions. This study identified specific areas that programs may focus on to improve fellow competency in the surgical management of epilepsy.

## Appendix Authors

**Table:**

Name	Location	Contribution
Hernan Nicolas Lemus, MD	Brigham and Women's Hospital, Boston, MA	Designed and conceptualized the study and drafted the manuscript for intellectual content
Barbara Dworetzky, MD	Brigham and Women's Hospital, Boston, MA	Critical interpretation of the data and edited the manuscript
Ellen J. Bubrick, MD	Brigham and Women's Hospital, Boston, MA	Critical interpretation of the data and edited the manuscript
Garth R. Cosgrove, MD	Brigham and Women's Hospital, Boston, MA	Critical interpretation of the data and edited the manuscript
Steven Tobochnik, MD	Brigham and Women's Hospital, Boston, MA	Designed and conceptualized the study and drafted the manuscript for intellectual content

## Glossary

ABPN: American Board of Psychiatry and Neurology
ACGME: Accreditation Council for Graduate Medical Education
CNP: clinical neurophysiology
DRE: drug-resistant epilepsy
NAEC: National Association of Epilepsy Centers

## References

[1] Brodie MJ, Barry SJE, Bamagous GA, Norrie JD, Kwan P. Patterns of treatment response in newly diagnosed epilepsy. Neurology. 2012;78(20):1548-1554. 22573629 10.1212/WNL.0b013e3182563b19 PMC3348850

[2] Engel J Jr. The current place of epilepsy surgery. Curr Opin Neurol. 2018;31(2):192-197. 29278548 10.1097/WCO.0000000000000528 PMC6009838

[3] Wiebe S, Blume WT, Girvin JP, Eliasziw M, Effectiveness and Efficiency of Surgery for Temporal Lobe Epilepsy Study Group. A randomized, controlled trial of surgery for temporal-lobe epilepsy. N Engl J Med. 2001;345(5):311-318. 11484687 10.1056/NEJM200108023450501

[4] Engel J Jr., McDermott MP, Wiebe S, et al, Early Randomized Surgical Epilepsy Trial ERSET Study Group. Early surgical therapy for drug-resistant temporal lobe epilepsy: a randomized trial. JAMA. 2012;307(9):922-930. 22396514 10.1001/jama.2012.220 PMC4821633

[5] Kaiboriboon K, Malkhachroum AM, Zrik A, et al. Epilepsy surgery in the United States: Analysis of data from the National Association of Epilepsy Centers. Epilepsy Res. 2015;116:105-109. 26310969 10.1016/j.eplepsyres.2015.07.007

[6] Engel J Jr. What can we do for people with drug-resistant epilepsy? The 2016 Wartenberg Lecture. Neurology. 2016;87(23):2483-2489. 27920283 10.1212/WNL.0000000000003407 PMC5177675

[7] Lowerison MW, Josephson CB, Jetté N, et al. Association of levels of specialized care with risk of premature mortality in patients with epilepsy. JAMA Neurol. 2019;76(11):1352-1358. 31380987 10.1001/jamaneurol.2019.2268 PMC6686748

[8] Ali M. The Epilepsy Milestone Project [online]. Accessed April 3, 2022. acgme.org/globalassets/pdfs/milestones/epilepsymilestones.pdf.

[9] ACGME. ACGME Program Requirements for Graduate Medical Education in Epilepsy [online]. acgme.org/globalassets/pfassets/programrequirements/184_epilepsy_2022_tcc.pdf.

[10] Albert D. Clinical Neurophysiology Milestones [online]. Accessed April 3, 2022. acgme.org/globalassets/PDFs/Milestones/ClinicalNeurophysiologyMilestones2.0.pdf?ver=2021-08-03-153737-587&ver=2021-08-03-153737-587.

[11] Daniello KM, Weber DJ. Education Research: the current state of neurophysiology education in selected neurology residency programs. Neurology. 2018;90(15):708-711. 29632112 10.1212/WNL.0000000000005296

[12] Nascimento FA, Gavvala JR. Education research: neurology resident EEG education: a survey of US neurology residency program directors. Neurology. 2021;96(17):821-824. 33310878 10.1212/WNL.0000000000011354

[13] Engel J Jr. Evolution of concepts in epilepsy surgery. Epileptic Disord. 2019;21(5):391-409. 31708489 10.1684/epd.2019.1091

[14] Ostendorf AP, Ahrens SM, Lado FA, et al. United States epilepsy center characteristics: a data analysis from the National Association of Epilepsy Centers. Neurology. 2022;98(5):e449-e458. 34880093 10.1212/WNL.0000000000013130 PMC8826463

[15] Weber D, McCarthy D, Pathmanathan J. An effective automated method for teaching EEG interpretation to neurology residents. Seizure. 2016;40:10-12. 27295562 10.1016/j.seizure.2016.05.009

[16] Diaz A, Sarac BA, Schoenbrunner AR, Janis JE, Pawlik TM. Elective surgery in the time of COVID-19. Am J Surg. 2020;219(6):900-902. 32312477 10.1016/j.amjsurg.2020.04.014 PMC7161516

[17] Benbadis SR. The tragedy of over-read EEGs and wrong diagnoses of epilepsy. Expert Rev Neurother. 2010;10(3):343. 20217964 10.1586/ern.09.157

[18] Amin U, Benbadis SR. The role of EEG in the erroneous diagnosis of epilepsy. J Clin Neurophysiol. 2019;36(4):294-297. 31274692 10.1097/WNP.0000000000000572

[19] Jackson JL, Kay C, Frank M. The validity and reliability of attending evaluations of medicine residents. SAGE Open Med. 2015;3:2050312115589648. 26770788 10.1177/2050312115589648 PMC4679281

[20] American Epilepsy Society. Self-assessments [online]. Accessed July 2022. aesnet.org/education/for-clinicians/self-assessment.

[21] American Epilepsy Society. AES Modules [online]. Accessed July 2022. aesnet.org/education/education/aes-emodules.

[22] American Board of Psychiatry and Neurology Epilepsy. Subspecialty Certification Examination in Epilepsy Medicine 2020 Content Blueprint [online]. Accessed July 2022. abpn.com/wp-content/uploads/2020/01/2020_Epilepsy_CERT_Content_Specifications.pdf.

[23] AES. EpiFITE Examination [online]. Accessed July 2022. aesnet.org/education/for-fellows/epifite.

[24] Thio LL, Edgar L, Ali I, et al. Epilepsy milestones 2.0: an updated framework for assessing epilepsy fellowships and fellows. Epilepsia. 2022;63(8):2155-2163. 35582760 10.1111/epi.17306

[25] Hamberger MJ, Williams AC, Schevon CA. Extraoperative neurostimulation mapping: results from an international survey of epilepsy surgery programs. Epilepsia. 2014;55(6):933-939. 24816083 10.1111/epi.12644 PMC4057949

[26] Karakis I, Liu L, Bensalem-Owen M, et al. Clinical neurophysiology fellowship program directors survey on a standardized fellowship match process: a call for action. J Clin Neurophysiol. 2021. 10.1097/WNP.0000000000000852 33878059

[27] Jehi L, Friedman D, Carlson C, et al. The evolution of epilepsy surgery between 1991 and 2011 in nine major epilepsy centers across the United States, Germany, and Australia. Epilepsia. 2015;56(10):1526-1533. 26250432 10.1111/epi.13116 PMC5082694

